# Hand-Portable Miniaturized Liquid Chromatography for the Determination of Chlorogenic Acids in Dietary Supplements

**DOI:** 10.3390/antiox11122408

**Published:** 2022-12-05

**Authors:** Camila Soto, Henry Daniel Ponce-Rodríguez, Jorge Verdú-Andrés, Pilar Campíns-Falcó, Rosa Herráez-Hernández

**Affiliations:** 1 MINTOTA Research Group, Departament de Química Analítica, Facultat de Química, Universitat de València, Dr. Moliner 50, 46100 Burjassot, Spain; 2 Departamento de Control Químico, Facultad de Química y Farmacia, Universidad Nacional Autónoma de Honduras, Ciudad Universitaria, Tegucigalpa 11101, Honduras

**Keywords:** dietary supplements, antioxidants, chlorogenic acids, miniaturized chromatography, portable liquid chromatography

## Abstract

With the explosive growth of the dietary supplements industry, new demands have emerged that cannot be faced with the sophisticated instrumentation available in well-equipped laboratories. In particular, there is a demand for simplified and easy-to-use instruments, capable of providing results in short times of analysis. In this study, a hand-portable miniaturized liquid chromatograph (portable LC) has been tested for the determination of chlorogenic acids (CGAs) in products intended to supplement the diet and elaborated with green coffee extracts. CGAs offer several health benefits due to their antioxidant properties, and an increasing number of dietary supplements are marketed with claimed high contents of these compounds. The results obtained with the proposed portable LC approach have been compared with those obtained with two other miniaturized benchtop liquid chromatography instruments, namely, a capillary liquid chromatograph (capLC) and a nano liquid chromatograph (nanoLC). Although compared with the methods that used the benchtop instruments, the sensitivity attainable was lower, the portable LC instrument provided a comparable analytical performance for the quantification of the main GCAs at low mg g^−1^ levels, and it was clearly superior in terms of speed. The proposed portable LC-based method can be applied to assess the content and distribution profile of the predominant CGAs in this kind of dietary supplement. It can be also used to estimate the antioxidant power due to CGAs, as well as their preservation state.

## 1. Introduction

Chlorogenic acids (CGAs) are naturally occurring phenolic compounds that can be found in many plants. From a chemical point of view, CGAs are esters of caffeic and quinic acids. The most abundant compounds are 5-caffeoylquinic acid or chlorogenic acid (5-CQA), 3-caffeoylquinic acid (3-CQA), 4-caffeoylquinic acid (4-CQA), 3,5-dicaffeoylquinic acid (3,5-CQA), 3,4-dicaffeoylquinic acid (3,4-diCQA), and 4,5-dicaffeoylquinic acid (4,5-diCQA) [1,2]. The content and distribution profile of CGAs depends not only on the type and part of the plant but also on external conditions such as exposure to UV radiation, and plant storage and processing [1].

In recent years, much attention has been paid to the content of CGAs in plants because of their remarkable antioxidant properties. The latest studies have shown the potential benefits of CGAs in the treatment of Alzheimer, diabetes, cardiovascular disease, and different types of cancer, as well as in fighting obesity [1,3,4]. As a result, there is a growing demand for plants with a high content of CGAs in the pharmaceutical, food, and cosmetic industries [5]. Green coffee beans are among the richest sources of CGAs, and an increasing number of dietary supplements containing green coffee extracts have been introduced into the market with several claimed benefits due to their antioxidant properties [6]. However, the regulations for the production and distribution of dietary supplements are not as strict as those set for pharmaceutical products [7,8]. In fact, dietary supplements are widely available not only in pharmacies and health stores but also in supermarkets and through e-commerce. Consequently, there is a growing concern about their quality and safety [9,10].

A variety of methods have been recently developed for the analysis of CGAs in different types of samples of botanical origin, with liquid chromatography (LC) being the technique most used. Examples are the work of Meinhart et al. who reported the determination of caffeic acid and the main CGAs in fruits using LC with UV-DAD detection [2], and the method developed by Prakash et al. for the quantification of 5-CQA (a compound typically used as a marker to establish the quality of natural products) in fruit pulp using ultra-performance LC with mass spectrometry (MS) [11]. Wang et al. reported the determination of 5-CQA in fermentation broths and fruits using LC with UV detection, comparing the results with those obtained using spectrophotometry with the reaction between this compound and potassium ferricyanide-Fe (III) [5]. The determination of the content and profile of CGAs in coffee beans has attracted great interest, as it is related to parameters such as the taste, aroma, antioxidant properties, and stability of coffee beverages, with LC with UV detection also as the preferred option [3,12,13]. A method based on LC-DAD-electrospray ionization-MS/MS has been developed to evaluate the influence of CGAs on the aroma and antioxidant properties of coffee brews [14], and LC-MS/MS has been used for the characterization of phytochemicals in Coffea arabica-derived samples [15]. Similar studies have been conducted using thin layer chromatography [16]. In those works, the analytes were extracted from the samples, typically with alcohol or water, and in some cases, using microwave- or ultrasound-assisted protocols [3,12]. However, none of them were applied to the analysis of dietary supplements. Very recently, an electrochemical method using screen-printed carbon electrodes modified with graphene and gold nanoparticles was proposed for the determination of the total content of CGAs in this kind of products [17]. Therefore, there is still a need for simple and rapid analytical tools for the determination of CGAs in dietary supplements that can be applied to assess their quality and safety or for a better characterization of the final products.

Miniaturized separation techniques have proved to be very useful in the identification and determination of several classes of compounds present in complex samples. Compared with conventional scale LC, miniaturized LC, such as capLC and nanoLC, provides enhanced resolution and detection capabilities; thus, it is increasingly used in many fields of application [18,19,20], including samples of a botanical origin [21]. However, in the analysis of dietary supplements, miniaturized LC has only been applied to a few amino acids, peptides, fatty acids, and flavonoids [22]. In a previous study, we have demonstrated the utility of capLC for the analysis of some terpenes [23], and more recently, we used both capLC and nanoLC for the analysis of caffeine in botanical dietary supplements [24]. Therefore, the potential of miniaturized chromatographic systems for the analysis of CGAs in this kind of product remains unexplored.

In the present work, we have evaluated the utility of a hand-portable miniaturized LC chromatograph for the determination of CGAs in dietary supplements elaborated from green coffee extracts. The results have been compared with those obtained with other miniaturized benchtop LC instruments, namely, a capillary chromatograph and a nano chromatograph; the latter instrument was coupled to an in-tube solid-phase microextraction (IT-SPME) device for online sample treatment [20,24]. The systems have been applied to the determination of the main CGAs in two green coffee extract-based dietary supplements. To the best of our knowledge, this is the first study on the utility of miniaturized LC and portable LC for the analysis of CGAs in dietary supplements.

## 2. Materials and Methods

### 2.1. Chemicals and Solutions

All reagents used throughout out the study were of analytical grade. Caffeic acid, caffeine, and formic acid (>98%) were obtained from Sigma-Aldrich (St. Louis, MO, USA); 5-CQA was purchased from Fisher Scientific (Pittsburgh, PA, USA), and 3-CQA, 4-CQA, 3,5-diCQA, 3,4-diCQA, and 4,5-diCQA were obtained from Phytopurify (Sichuan, China). Hydrochloric acid (37%) and phosphoric acid (85%) were supplied by Scharlau (Barcelona, Spain). HPLC grade methanol and acetonitrile were purchased from VWR Chemicals (Randnor, PA, USA). Stock solutions of the analytes (1000 µg mL^−1^) were prepared in methanol. Working solutions of the analytes and their mixtures were prepared by diluting the stock solutions with water. All solutions were stored in the dark at 4 °C until use.

Ultrapure water was obtained from an Adrona system (Riga, Latvia). Water was filtered through 0.22 µm nylon membranes purchased from GVS (Sandfor, ME, USA) before use.

### 2.2. Chromatographic Conditions

The capLC system consisted of a capillary pump (Agilent 1100 Series, Waldbronn, Germany) equipped with a Rheodyne model 7725 six-port injection valve, a 12 µL injection loop, and a photodiode array detector (Agilent 1200 Series). An Agilent HPLC ChemStation system was used for data acquisition and calculation. The column installed was a Zorbax SB C18 (150 mm × 0.5 mm id, 5 µm) (Agilent). The mobile phase was a mixture of water with 0.1% phosphoric acid and methanol in gradient elution. The percentage of methanol was increased from 25% (*v*/*v*) at zero min to 30% at 7 min to 50% at 15 min, kept constant until 16 min, and then increased to 75% at 20 min, and to 100% at 23 min. The flow rate was 12 µL min^−1^. The chromatograms were recorded between 190 and 400 nm and monitored at 330 nm.

The benchtop nanoLC system was an Agilent 1260 Infinity nanoLC chromatograph equipped with a 10-port electronically controlled switching valve (Valco Instruments, Houston, TX, USA) and a UV-DAD detector with an 80 nL nanoflow cell (Agilent). The detector was coupled to a data system (Agilent, ChemStation) for data acquisition and treatment. The chromatograms were recorded between 190 and 400 nm and monitored at 330 nm. The separative column was a Zorbax 300SB C18 (150 mm × 100 µm i.d., 3.5 µm particle size) column (Agilent). For the online enrichment of the CGAs, an IT-SPME device proposed previously for the analysis of caffeine was used. A detailed description of such a device can be found in [24]. Portions of 100 µL of the working solutions were loaded in the extractive capillary of the IT-SPME device using a 250 µL precision syringe. Next, the capillary was flushed with 100 µL of water to remove unwanted matrix components, and the analytes were transferred to the separative column with the mobile phase by rotating the switching valve. The mobile phase was a mixture of water containing 0.1% formic acid (*v*/*v*) and acetonitrile. The percentage of acetonitrile in the mobile phase was linearly increased from 7% at 0 min to 10% at 1 min and to 45% at 2 min; then, it was kept constant until 4 min, increased to 70% at 5 min, kept constant until 7 min, and increased to 80% at 8 min. Finally, the percentage of acetonitrile was increased to 95% at 11 min and then kept constant until the end of the run. The flow rate was 0.5 µL min^−1^.

For assays with the portable LC system, a portable Axcend Focus LCTM LC liquid chromatograph (Axcend Corp, Provo, UT, USA) was used. The separation was carried out in a 100 mm × 150 µm i. d. column packed with fused silica particles, 1.7 µm particle size. On-capillary UV absorbance was measured at 255 nm using a LED. The volume of the internal injector loop was 40 nL, whereas aliquots of about 10 µL of the samples were loaded in the injection port using a 25 µL syringe. An Axcend Focus v2.0 software was used for data treatment. The eluent was a mixture of solvent A (water with 3% of acetonitrile, *v*/*v*) and solvent B (acetonitrile with 3% of water, *v*/*v*). The percentage of B was increased from 5% at 0 min to 20% at 4 min to 60% at 4.5 min and kept constant until 5.5 min. Next, the percentage of B was increased to 95% at 6.0 min and kept constant until the end of the run (6.5 min). The flow rate was 2 µL min^−1^.

All of the solvents were filtered using 0.22 µm nylon membranes (GVS).

### 2.3. Analysis of the Real Samples

The two dietary supplements used in the study were acquired in local supermarkets. According to the labels, one of the samples (sample A) contained 437 mg of decaffeinated green coffee extract per gram of sample, with the amount of GCAs 198 mg per g of sample. The other sample (sample B) contained 400 mg of green coffee extract, 100 mg of green tea extract, and 100 mg of bitter orange as main ingredients; the amount of CGAs was 200 mg per g of sample.

Ultrasound-assisted extraction with methanol was the option selected for the isolation of the compounds of interest, using a procedure previously optimized for the analysis of terpenes in different types of dietary supplements of botanical origin [23]. Briefly, the samples (≈25 mg) were vortexed with 5 mL of methanol for 1 min and then treated in an ultrasonic bath (300 W, 40 kHz, Sonitech, Guarnizo, Spain) for 5 min. Next, the liquid phase was removed and filtered with 0.22 µm nylon membranes. Finally, 90 µL of the filtered extracts were mixed with 10 µL of 0.1% hydrochloric acid (*v*/*v*). If required, the extracts were previously diluted with water.

All the experiments were carried out at room temperature, which ranged from 19 to 24 °C throughout the study.

## 3. Results

### 3.1. Chromatographic Conditions

**Figure 1:**
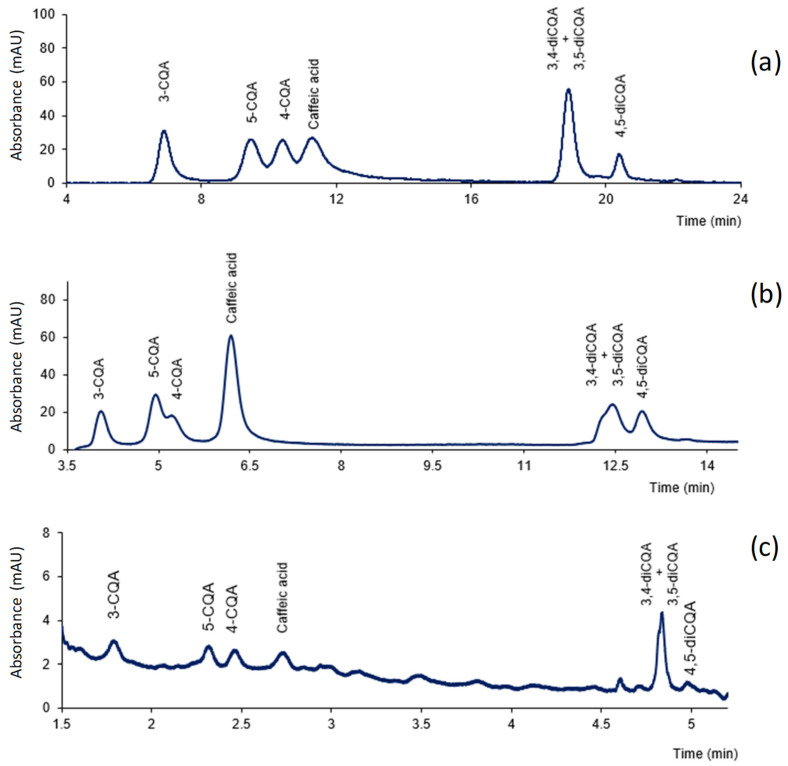
Chromatograms obtained for standard mixtures of caffeic acid and CGAs under the proposed conditions: (**a**) chromatogram obtained at 330 nm for a solution containing 1 µg mL^−1^ of each compound with the capLC system; (**b**) chromatogram obtained at 330 nm for a solution containing 0.5 µg mL^−1^ of each compound with the benchtop nanoLC system; (**c**) chromatogram obtained at 255 nm for a solution containing 60 µg mL^−1^ with the portable LC system. For other experimental details, see text.

Initially, different mobile-phase flow rates and elution programs were assayed with the three chromatographic systems tested, using standard solutions of the individual CGAs and their mixtures. The aim of this study was to find the conditions suitable for the analysis of CGAs with the three systems operating under typical reversed phase conditions. The mobile phases used in the study were mixtures of acetonitrile or methanol and water; with the benchtop capLC and nanoLC instruments, different modifiers were added to reduce peak tailing. With the capLC system, the best results were obtained when using methanol as the organic solvent and phosphoric acid as the modifier. However, eluents with methanol were not suitable for the nanoLC instrument because they produced system pressures which were too high for the IT-SPME device. In the nanoLC system, the best chromatographic profiles were found when using acetonitrile and water containing formic acid. Finally, for the portable instrument, suitable resolution was obtained using water–acetonitrile, which was then the eluent selected. A suitable resolution was observed in most of the conditions assayed except for 3,4-diCQA and 3,5-diCQA, which overlapped severely regardless of the chromatographic system and elution program used. Those observations can be explained by the fact that both compounds have very similar chemical structures [1]. Moreover, the two compounds exhibited almost identical UV spectra (Figure S1). In view of those results, the elution conditions were selected to obtain the suitable separation of the other compounds in the minimum time of analysis. In Figure 1 are shown the chromatograms obtained under the conditions finally selected for a mixture of the analytes with the three chromatographic systems tested. As can be observed, 3,4-diCQA and 3,5-diCQA eluted as a single peak in the capLC system, whereas two severely overlapped peaks were found with the benchtop nanoLC and portable LC systems. The runtimes were in agreement with the characteristics of the three systems (column dimensions, flowrates, detector cells) and were considerably lower with the portable LC chromatograph [24].

### 3.2. Stability of CGAs

**Figure 2:**
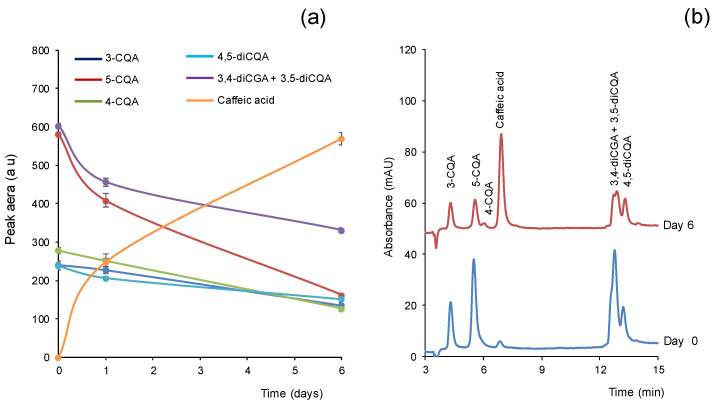
(**a**) Variation in the peak areas of CGAs in a solution freshly prepared and after storage. Concentration of the analytes, 0.5 µg mL^−1^ each; storage conditions, 4 °C in the dark. (**b**) Chromatograms obtained at 330 nm with the benchtop nanoLC system for a solution of CGAs freshly prepared and the same solution after storage for 6 days. For other details, see text.

In the course of our studies with standard solutions of the analytes, we observed a significant decrement in their responses (peak area) with time, even in solutions kept at 4 °C and in the dark. A decrease was found for all of the analytes, although it was more pronounced for 5-CGA. This is illustrated in Figure 2a, which shows the variation in the peak areas obtained for a solution over time. As observed, the day after the preparation of the solution, the peak area obtained for 5-CGA decreased by about 30% with respect to the freshly prepared solution. After six days, the peak area obtained for 5-CGA was only around a fourth part of the initial value. In parallel with the decrement in the peaks of the analytes, an additional peak at a retention time of 7.0 min was observed. This is illustrated in Figure 2b, which shows the chromatograms obtained with the benchtop nanoLC system for a standard solution containing a mixture of the tested compounds freshly prepared and 6 days after. The peak at 7.0 min was identified as caffeic acid, which is in agreement with the results reported by other authors [25,26]. As this compound could be resolved from those of the CGAs under the three tested chromatographic systems (see Figure 1), it was included in the study as a marker to detect the possible degradation of the CGAs.

### 3.3. Analytical Performance

The analytical performance of the three methods was evaluated using standard solutions of the analytes freshly prepared. With the benchtop chromatographs, the peak areas were measured at the maximum of the spectra of the CGAs, which was around 330 nm; caffeic acid presented a similar spectrum (Figure S1). The portable LC system only provided the chromatograms at 255 nm.

The linearity was evaluated by processing in duplicate solutions of the analytes at five concentrations within the tested ranges. The limits of detection (LODs) were calculated as the concentrations of analytes that resulted in signal-to-noise ratios of 3. These values were established through the successive injection of solutions with decreasing concentrations of the analytes. Water was processed between those solutions to confirm the absence of contaminants and/or memory effects. Finally, the precision was established by injecting three replicates of the standard solutions of the analytes at half of the highest concentration assayed. The values obtained are summarized in Table 1.

As observed from Table 1, the linearity was suitable for all of the compounds tested with the three chromatographic systems. As expected, the two benchtop systems provided better sensitivity (higher slopes of the calibration equations) and lower LODs, as the portable LC instrument did not provide the chromatograms at the wavelength corresponding to the absorption maximum of the CGAs. With regards the two benchtop instruments, the LODs attainable with the nanoLC chromatograph were lower than those found with the capLC, which can be mainly explained by the online enrichment produced by the IT-SPME device. It has to be noted that the slopes of the calibration equations obtained for 3,4-diCQA and 3,5-diCQA were rather similar in the three systems. Therefore, the area obtained for the peak (or peaks) observed at their retention times could be used as an estimation of the total amount of both compounds in the working solution.

The precision obtained with the three methods was also satisfactory; the relative standard deviations (RSDs) were ≤8% (n = 3). Moreover, no significant differences were found in the values obtained by the three chromatographic approaches tested.

### 3.4. Extraction Recoveries and Stability of the Extracts

For the extraction of the target compounds, an ultrasound-assisted extraction protocol previously developed for samples of botanical origin was applied [23]. It has to be remarked that the extracts were processed immediately after being obtained because, as for the standard solutions, an important degradation of the CGAs was observed, with the subsequent increment in the peak due to caffeic acid (see Figure S2).

The recoveries were calculated by spiking one of the samples (sample B) with known quantities of the CGAs and using the capLC method. The amount of each compound added to the sample was 5 µg g^−1^. The analytes were extracted from the samples (25 mg) with 5 mL of methanol using an ultrasonic bath. The recoveries were calculated by comparing the increments in the peak areas in the spiked samples and the areas obtained for standard solutions containing concentrations of the analytes equivalent to the concentrations added to the sample. The recoveries obtained under the proposed conditions ranged from 85 to 90%, as summarized in Table 2. Although not tested, a higher overall recovery of the CGAs could be achieved by treating the sample residue with a second portion of methanol [23]. However, this would result in a lower concentration of CGAs in the combined extract. Thus, an additional step aimed at the partial or total evaporation of the extracts would be necessary to avoid a loss of sensitivity in the portable LC method. Considering that this method was proposed as a simple and fast alternative for the analysis of CGAs, a sample treatment involving re-extraction and evaporation steps was discarded.

To obtain the intra-day precision of the entire procedure, three portions of the sample were spiked with the analytes and processed. The inter-day precision was established from the results obtained for six spiked samples processed on different days. The results are also listed in Table 2. According to the results, the RSD values were similar to those found for the standard solutions (Table 1).

### 3.5. Application to the Quantification of CGAs in Dietary Supplements

Finally, the proposed methods were applied to the analysis of the extracts obtained from two dietary supplements containing green coffee extract as the main active ingredient. Portions of 25 mg of the samples were extracted using 5 mL of methanol. The extracts were then treated with 0.1% hydrochloric acid and chromatographed. With the capLC and nanoLC methods, a previous dilution of the extracts was necessary to adjust the concentration of the analytes to their respective linear intervals (Table 1); water was used for diluting the extracts.

**Figure 3:**
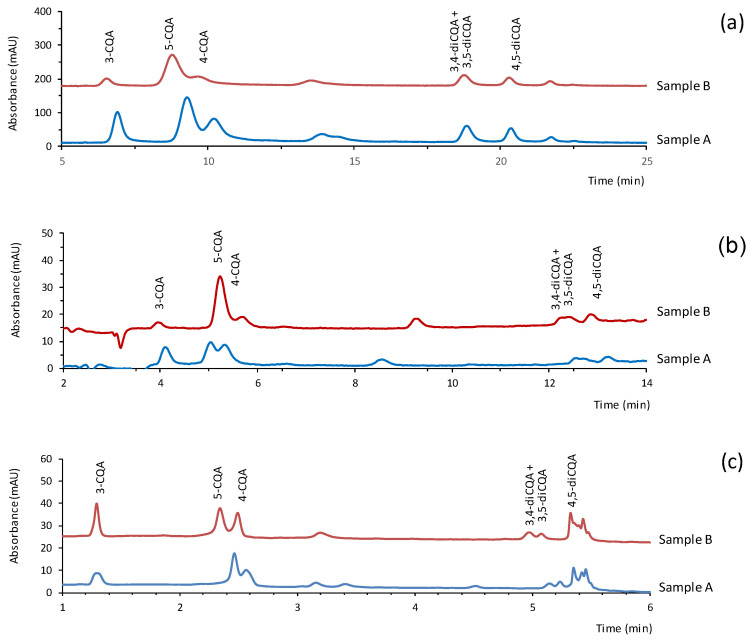
Chromatograms obtained for the samples with the three miniaturized LC systems tested: (**a**) chromatograms obtained at 330 nm with the capLC; (**b**) chromatograms obtained at 330 nm with the benchtop nanoLC system; (**c**) chromatograms obtained at 255 nm with the portable LC. The dilution factors for the samples are those listed in Table 3. For other experimental details, see text.

The registers obtained for the samples with the three miniaturized systems are shown in Figure 3. As expected, the two samples contained significant amounts of CGAs. Besides the peaks of the CGAs included in this study, minor peaks were observed within the time window defined by the peaks of 4-CGA and 3,4-diCGA + 3,5-diCGA. Those minor peaks exhibited UV spectra similar to those of the CGAs (Figure S1). It is worth mentioning that, although a large number of CGAs can be found in plants, the compounds included in most of the methods reported in the literature are the same as those tested in the present work. This can be explained not only by the fact that they are the most abundant CGAs in coffee but also because of the lack of commercially available standards for other members of the family that may be present at trace levels [1,12,16]. Small peaks of caffeic acid were detected in the samples only with the benchtop nanoLC method.

The concentrations of the analytes in the extracts were calculated by interpolating the peak areas obtained for the analytes in the calibration equations of Table 1. Those concentrations were transformed into amounts of CGAs in the samples, taking into account the dilution factors (if applicable) and the recovery values of Table 2. The results obtained are summarized in Table 3. In this table, the total contents of 3,4-diCQA and 3,5-diCQA (calculated as 3,5-diCQA) are given.

As it can be deduced from Table 3, the results obtained for the samples using the three miniaturized LC approaches were rather similar. The differences between the values found by capLC and the portable LC methods led to a mean value statistically equivalent to zero for a 95% confidence level (t_calculated_ = 1.070, t_theoretical_ = 2.262). Similar results were found for the differences between the values obtained by the benchtop nanoLC and portable LC methods (t_calculated_ = 0.205). It was concluded that the portable LC method provided results statistically equivalent to those obtained with the methods that used the benchtop instruments.

The results of Table 3 also show that the relative percentages of the individual compounds varied between the two samples tested. For example, in sample A, the percentages of the (mono)caffeoylquinic acid derivatives were higher than those of the dicaffeoylquinic derivatives, whereas in sample B, the concentration of all of the tested were comparable with the exception of 5-CGA, which was significantly higher. The content of 5-CGA in this sample was also higher than in sample A. The presence of a different source of CGAs (green tea extract) among its ingredients may be the reason for the difference.

According to their labels, the content of CGAs in products A and B were 198 and 200 mg per g, respectively. The results of Table 3 show that the total amounts of CGAs found for sample B with the three chromatographic methods used was slightly lower than the declared value. The small difference between the value found and the declared value can be explained by the small quantities of the CGAs not included in the study; it may also be due to a slight degradation of the sample, which is compatible with the presence of a small amount of caffeic acid in the extracts. However, the value found for sample A was much lower than the declared value. This discrepancy suggested that the content of CGAs in the sample was measured using a different analytical approach. It has to be noted that the spectroscopic methods proposed for the determination of CGAs are typically based on the measurement of the total absorbance of the extracts, directly or after a chemical reaction, and the results are expressed as 5-CQA [5,27,28]. The values obtained with such methods may be different from those derived from the analysis of the individual CGAs. Moreover, the declared values may refer to the raw plants used for preparing the dietary supplements. Thus, those values would not reflect the possible alterations in the CGA content during the production and storage of the final product [1,13,14]. In this respect, the three methods applied in the present study provided a more reliable characterization of the samples.

In the course of our study (≈one year), we did not observe variations in the percentages of the CGAs in the dietary supplements tested.

## 4. Discussion

The widespread consumption of dietary supplements has led to an increasing demand for analytical results in order to ensure their quality and safety. Instruments capable of providing information on the chemical composition of this type of products in short times of analysis are highly desirable in laboratories that must deal with a large amount of samples [22]. In this context, the main advantage of the portable LC method developed for the determination of CGAs is its speed. To the best of our knowledge, only a method has been specifically developed for the fast analysis of CGAs in green-coffee extracts, but it was based on a benchtop conventional-scale LC [12]. The results of the above sections have shown that the proposed portable LC method allows the determination of the main CGAs of green coffee extract-based dietary supplements in a few min. The inherent simplicity of this system, especially with regard to the detector, can be a limitation in terms of sensitivity when compared with the benchtop capLC and nanoLC instruments. In addition, the benchtop systems are better suited for online treatments aimed at enhancing the selectivity and/or sensitivity. This has been illustrated in the present study by coupling IT-SPME to the nanoLC instrument. Nevertheless, the proposed portable LC method allowed the determination of the main CGAs in the tested products, with the results obtained statistically comparable to those found with the methods that used the benchtop instruments. The portable LC approach can be also used to obtain information about the distribution profile of the predominant CGAs. Due its speed and ease of use, the portable LC method can be an alternative to the spectroscopic methods proposed to estimate the total content of CGAs, which typically involve chemical reactions to form colored species [5].

It has to be noted that portable miniaturized LC instruments have been recently introduced into the market. Although their prices are comparable to those of benchtop chromatographs, the costs derived from the consumption of power and mobile-phase solvents are significantly reduced. The other relevant advantages of the portable LC instrument are that, because of their simplicity, they can be operated by personnel with a basic knowledge of chromatography principles, and laboratories with sophisticated facilities are not required. In addition, although in the present study the instrument was used under laboratory conditions, it can be used for on-site analysis making unnecessary to transport the samples to the laboratory.

Although we only applied the proposed method to the analysis of final products, the variation in the content of chlorogenic acids during industrial processes may be of interest [5]. Due to its characteristics, if required, the portable LC method could be adapted to the analysis of CGAs in dietary supplements in other contexts (control of raw materials, production, or distribution).

## 5. Conclusions

In this work, we have developed a method for the analysis of CGAs in dietary supplements using a hand-portable LC instrument. The results obtained have been compared with those provided by other benchtop miniaturized instruments which have been used as reference methods, namely, a capLC chromatograph and a nanoLC chromatograph. To the best of our knowledge, this is the first study that shows the application of a miniaturized LC for the analysis of CGAs in dietary supplements.

The results obtained with the hand-portable LC instrument were comparable in terms of accuracy and precision to those achieved with the benchtop instruments for the quantification of CGAs at mg g^−1^ levels, although the sensitivity attainable was lower. The main advantage of the proposed method in comparison with those using the benchtop instruments is that the time of analysis is significantly lower. It can be concluded that the portable LC-based method is a reliable option to reduce the workload of laboratories dealing with the analysis of dietary supplements. The present study has also shown the impact that the time elapsed between the sample pretreatment and the measurement step may have on the analytical results.

## Figures and Tables

**Table 1:** Analytical parameters of the three tested methods.

System	Compound	Linearity, y = ax + b (n = 10)				LOD (µg mL^−1^)	Precision *, (n = 3) RSD (%)
		Concentration Range (µg mL^−1^)	a ± *s*_a_	b ± *s*_b_	R^2^		
CapLC	3-CQA	0.2–2.0	1180 ± 20	−80 ± 20	0.998	0.05	6
	5-CQA	0.2–2.0	1160 ± 30	−110 ± 30	0.996	0.05	2
	4-CQA	0.3–2.0	1200 ± 20	−200 ± 30	0.998	0.1	5
	3,4-diCQA	0.3–2.0	650 ± 40	−90 ± 40	0.990	0.1	6
	3,5-diCQA	0.3–2.0	670 ± 30	−20 ± 30	0.990	0.1	4
	4,5-diCQA	0.3–2.0	560 ± 20	−60 ± 20	0.993	0.1	8
	Caffeic acid	0.3–2.0	2300 ± 100	−600 ± 100	0.993	0.1	8
anoLC	3-CQA	0.01–0.1	643 ± 11	4416	0.998	0.005	2
	5-CQA	0.01–0.1	1151 ± 17	2 ± 1	0.9990	0.005	5
	4-CQA	0.025–0.2	650 ± 9	−9 ± 1	0.9990	0.010	5
	3,4-diCQA	0.010–0.1	479 ± 8	2 ± 1	0.998	0.005	5
	3,5-diCQA	0.01–0.1	483 ± 9	2 ± 1	0.998	0.005	5
	4,5-diCQA	0.01–0.1	576 ± 11	1 ± 1	0.998	0.005	1
	Caffeic acid	0.01–0.5	1920 ± 5	−40 ± 10	0.992	0.005	1
Portable LC	3-CQA	30–80	0.134 ± 0.007	−2.1 ± 0.3	0.98	10	1
	5-CQA	30–80	0.108 ± 0.003	−1.1 ± 0.1	0.994	5	1
	4-CQA	20–80	0.106 ± 0.003	−0.28 ± 0.08	0.994	5	2
	3,4-diCQA	20–80	0.161 ± 0.007	−0.64 ± 0.17	0.993	5	2
	3,5-diCQA	20–80	0.169 ± 0.006	−0.43 ± 0.15	0.995	5	2
	4,5-diCQA	20–100	0.204 ± 0.007	−0.71 ± 0.17	0.990	5	1
	Caffeic acid	20–80	0.313 ± 0.010	−4.8 ± 0.5	0.993	5	2

* Established at concentration of 1 µg mL^−1^ with the capLC method, 0.1 µg mL^−1^ for 4-CGA, 0.05 µg mL^−1^ for the rest of compounds using the nanoLC method, and 50 µg mL^−1^ using the portable LC method.

**Table 2:** Precision and recovery obtained in a spiked sample (5 mg g^−1^ of each compound) with the capLC method.

Compound	Mean Recovery (n = 6) (%)	Precision, RSD (%)	
		Intra-Day (n = 3)	Inter-Day (n = 6)
3-CQA	87	3	6
5-CQA	85	5	8
4-CQA	90	2	6
3,4-diCQA	87	1	1
3,5-diCQA	89	3	2
4,5-diCQA	89	3	2
Caffeic acid	85	3	7

**Table 3:** Results obtained for the real samples analyzed (n = 3).

Sample	Compound	Amount Found in the Sample (mg g^−1^)					
		CapLC		NanoLC		Portable LC	
		Dilution	Mass	Dilution	Mass	Dilution	Mass
A	3-CQA	1:300	21.0 ± 0.3	1:500	25.6 ± 0.3	Not applicable	21.0 ± 0.4
	5-CQA		33 ± 3		23.6 ± 0.4		43 ± 1
	4-CQA		23 ± 2		37 ± 1		27 ± 1
	3,4-diCQA + 3,5-diCGA		9.3 ± 0.5		11 ± 1		9 ± 1
	4,5-diCQA		12.0 ± 3.0		13 ± 2		14 ± 1
	Total:		98		110		114
B	3-CQA	1:300	12.2 ± 0.8	1:2000	18.6 ± 0.4	Not applicable	17 ± 2
	5-CQA		78 ± 2		91 ± 2		101 ± 8
	4-CQA		20 ± 2		23 ± 2		20 ± 1
	3,4-diCQA + 3,5-diCGA		33 ± 2		35 ± 2		27 ± 1
	4,5-diCQA		28 ± 2		27 ± 4		20 ± 1
	Total:		171		195		185

## Data Availability

Data is contained within the article and Supplementary Materials.

## Supplementary Materials

The following supporting information can be downloaded at: https://www.mdpi.com/article/10.3390/antiox11122408/s1, Figure S1: UV spectra of the tested compounds registered at the retention times of the analytes with the capLC system; Figure S2: Chromatograms at 330 nm obtained with the benchtop nanoLC system for the extract of sample A and the same extracts 20 days after being kept at 4 °C in the dark.
