# Supplementary material for: Hand-Portable Miniaturized Liquid Chromatography for the Determination of Chlorogenic Acids in Dietary Supplements
Source: Antioxidants (Basel). 2022 Dec 5;11(12):2408. doi: 10.3390/antiox11122408 (PMC9774231; doi:10.3390/antiox11122408)
Supplement: Supplementary file 1 [file antioxidants-11-02408-s001.zip › antioxidants-2041136-supplementary.pdf]

Supplementary content

Portable nano-liquid chromatography for nutraceuticals

C. Soto[1], H. D. Ponce-Rodríguez[1,2] (ORCID:0000-0002-8949-7679), J. Verdú-Andrés[1] (ORCID: 0000-0002-7261-4488), R. Herráez-Hernández[1*] (ORCID 0000-0002-5106-0826), P. Campíns-Falcó[1*] (ORCID: 0000-0002-0980-8298)

1- MINTOTA Research Group, Departament de Química Analítica, Facultat de Química, Universitat de València, Dr. Moliner 50, 46100-Burjassot, València, Spain.

2- Departamento de Control Químico, Facultad de Química y Farmacia, Universidad Nacional Autónoma de Honduras, Ciudad Universitaria, Tegucigalpa, Honduras.

*Corresponding author: Tel.:34-94-3544978; Fax: 34-94-3544436

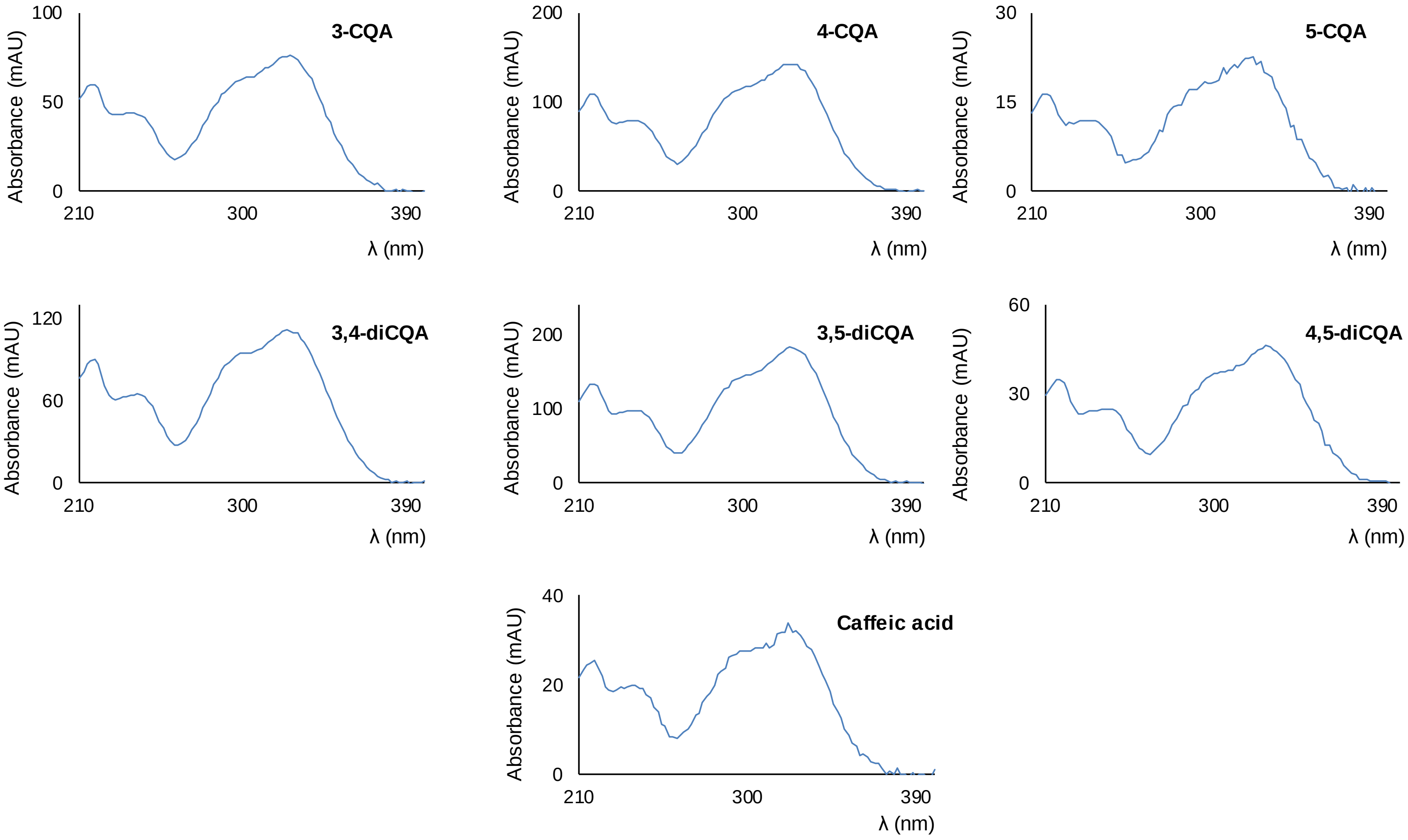

Figure S1. UV spectra of the tested compounds registered at the retention times of the analytes with the capLC system.

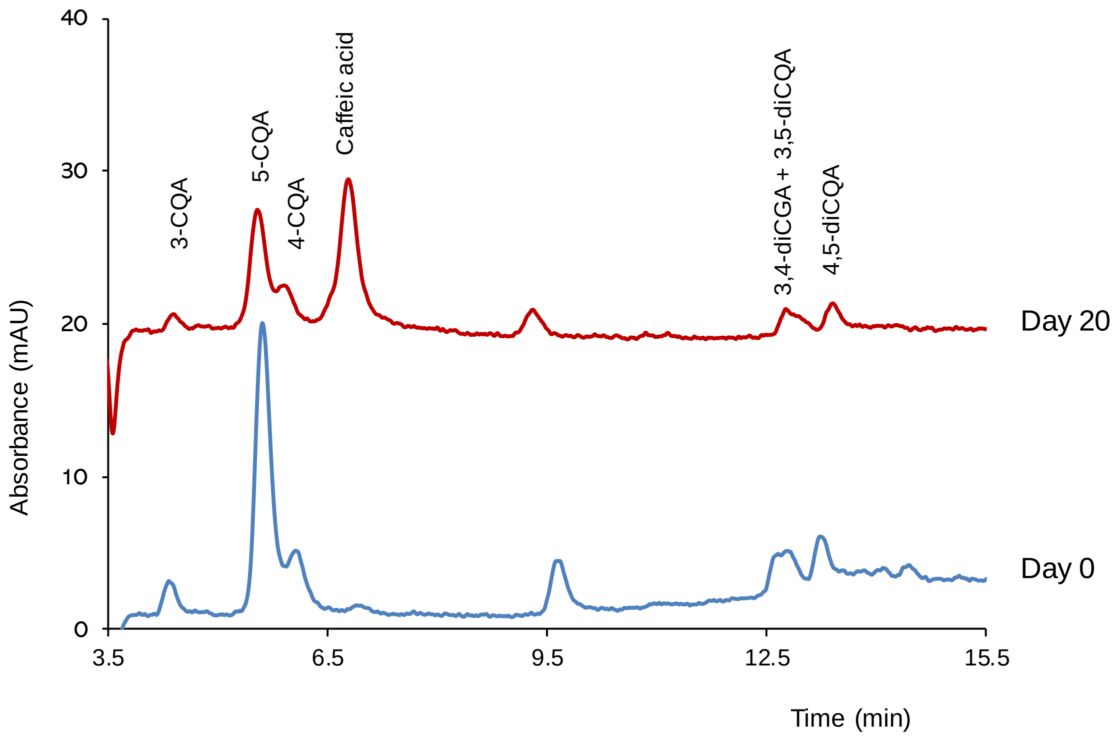

Figure S2. Chromatograms at 330 nm obtained with the benchtop nanoLC system for the extract of sample A, and the same extracts 20 days after being kept at 4 ^{0}C in the dark.
